# Prehospital Tourniquets in Civilians: A Systematic Review

**DOI:** 10.1017/S1049023X20001284

**Published:** 2021-02

**Authors:** Kenneth A. Eilertsen, Morten Winberg, Elisabeth Jeppesen, Gyri Hval, Torben Wisborg

**Affiliations:** 1. University of Oslo, Oslo, Norway; 2. Faculty of Health Sciences, University of Stavanger, Stavanger, Norway; 3. Department of Research, The Norwegian Air Ambulance Foundation, Oslo, Norway; 4. Norwegian Institute of Public Health, Oslo, Norway; 5. University of Tromsø, The Arctic University of Norway, Hammerfest, Norway; 6. Dept. of Anaesthesia and Intensive Care, Hammerfest Hospital, Finnmark Health Trust, Hammerfest, Norway; 7. Norwegian National Advisory Unit on Trauma, Division of Emergencies and Critical Care, Oslo University Hospital, Oslo, Norway

**Keywords:** first responder, hemorrhage, penetrating wounds, tourniquet

## Abstract

**Objectives:**

Terrorist attacks and civilian mass-casualty events are frequent, and some countries have implemented tourniquet use for uncontrollable extremity bleeding in civilian settings. The aim of this study was to summarize current knowledge on the use of prehospital tourniquets to assess whether their use increases the survival rate in civilian patients with life-threatening hemorrhages from the extremities.

**Design:**

Systematic literature review in Medline (Ovid), Embase (Ovid), Cochrane Library, and Epistemonikos was performed using the Preferred Reporting Items for Systematic Reviews and Meta-Analyses (PRISMA) Guidelines. The search was performed in January 2019.

**Setting:**

All types of studies that examined use of tourniquets in a prehospital setting published after January 1, 2000 were included.

**Primary/Secondary Outcomes:**

The primary outcome was mortality with and without tourniquet, while adverse effects of tourniquet use were secondary outcomes.

**Results:**

Among 3,460 screened records, 55 studies were identified as relevant. The studies were highly heterogeneous with low quality of evidence. Most studies reported increased survival in the tourniquet group, but few had relevant comparators, and the survival benefit was difficult to estimate. Most studies reported a reduced need for blood transfusion, with few and mainly transient adverse effects from tourniquet use.

**Conclusion:**

Despite relatively low evidence, the studies consistently suggested that the use of commercial tourniquets in a civilian setting to control life-threatening extremity hemorrhage seemed to be associated with improved survival, reduced need for blood transfusion, and few and transient adverse effects.

## Introduction

Trauma in the civilian setting may differ from trauma in the military, but the last decades’ terrorist attacks and mass-casualty events world-wide have made penetrating injuries more similar in the two settings than before. Therefore, knowledge from both settings can be valuable when informing national guidelines. This new panorama of injuries, often with multiple casualties occurring at the same time, has forced a re-evaluation of treatment and first responder recommendations. In the US, early hemorrhage control has become a central focus for improving survival in life-threatening extremity bleeding following the Hartford Consensus.^1–4^ They recommend that civilian bystanders, law enforcement officers, and Emergency Medical Services (EMS) personnel be equipped with and use tourniquets.

In the last few years, Europe has had numerous terrorist attacks and mass-casualty events, including Oslo/Utøya 2011, Paris 2015, Nice 2016, and Berlin 2016. After the July 22, 2011 attacks in Norway,^5^ the Norwegian Directorate of Health (Oslo, Norway) published a report in which one of the recommendations was to establish updated national guidelines for the use of tourniquets by police officers. Few countries have coherent national guidelines for the use of tourniquets. Some systematic reviews already exist on the subject, but few had sufficiently broad search strategy, were published in English, and included both military and civilian studies. A new systematic review was therefore considered necessary.

This systematic review aims to summarize current knowledge of the use of prehospital tourniquets to be used as a theoretical framework for developing guidelines for prehospital treatment in civilian settings. The primary outcome was to assess whether the use of prehospital tourniquets increases the survival rate in civilian patients with life-threatening hemorrhage from the extremities. Secondary outcomes were number of transfusions, complications, and other adverse events, if available.

## Methods

To conduct this systematic review, the Preferred Reporting Items for Systematic Reviews and Meta-Analysis (PRISMA) Guidelines^6^ were utilized (checklist is included in Appendix I; available online only). The protocol was published on PROSPERO (ID-number: CRD42019123172).

### Differences between Protocol and Review

As described in the Introduction, penetrating injuries in civilian settings are more similar to such injuries in military settings now than they were before. Studies in military settings were therefore included. It was suspected that there would be few controlled studies on this intervention, and all types of study designs were identified. However, only studies with more than 20 cases were included in the primary outcome (mortality). All studies were included when assessing the secondary outcomes (blood transfusions, complications of the extremity, or other adverse events) to identify rare complications. These decisions were made before the search was done and before the inclusion process.

### Inclusion Criteria

To identify all relevant studies on the topic, the following inclusion criteria were used: (1) *Population*: Adult patients aged ≥16 with life-threatening hemorrhages from the extremities; (2) *Intervention*: Treatment with tourniquet by professionals or laymen; (3) *Comparison*: Hemorrhage control with other measures; (4) *Outcomes*: Mortality, number of blood transfusions, complications of the extremity, and other adverse events - studies had to report on patient outcomes; (5) *Study Design*: All studies with more than 20 informants for primary outcome and all studies for secondary outcomes; (6) *Language*: All languages that could be translated by automatic translation engines were included, however, Cyrillic and Chinese papers were excluded; and (7) *Publication Year*: Studies published from 2000 onwards.

### Literature Search

The following electronic databases were searched for eligible studies on January 10, 2019: Medline (Ovid; US National Library of Medicine, National Institutes of Health; Bethesda, Maryland USA), Embase (Ovid; Elsevier; Amsterdam, Netherlands), Cochrane Library (The Cochrane Collaboration; London, United Kingdom), and Epistemonikos (Epistemonikos Foundation; Santiago, Chile). The search was limited to studies published from 2000 onwards. PROSPERO, clinicaltrials.gov, and World Health Organization International Clinical Trials Registry Platform (WHO ICTRP; Geneva, Switzerland) were searched for planned and on-going studies. The search was peer-reviewed and is documented in Appendix II (available online only).

In addition to the electronic search, the reference lists of included studies and systematic reviews were searched.

### Study Selection and Data Extraction

The records identified from different sources were collated into a systematic review screening tool (RAYYAN; Qatar Computing Research Institute; Doha, Qatar)^7^ in which duplicates were removed. References obtained from database and literature searches were independently examined at the title/abstract level by two authors, with discrepancies resolved by consensus, and then retrieved as complete articles if determined to be potentially pertinent. The studies were included if they met the inclusion criteria. The search results were exported to EndNote (Clarivate; Philadelphia, Pennsylvania USA).^8^

Some of the studies reported data from the same population/database. In that case, only data extracted from the latest published study with the largest sample size that reported relevant outcomes were used.

### Quality Appraisal

The Critical Appraisal Skills Programme (CASP; Oxford, United Kingdom) checklist for critical appraisal of observational studies was employed.^9^ The critical appraisal was done by KAE and MW, and discrepancies resolved through discussion. No randomized controlled studies were identified; therefore, all relevant published material was systematically reviewed - independent of study design. Case reports were only included to assess secondary outcomes. The reliability of the evidence was assessed using the Grading of Recommendations Assessment, Development, and Evaluation (GRADE) approach.^10^ The certainty of the evidence was rated as low or very low due to the observational designs, high risk of bias, and uncertain “dose-responds.”

### Definitions

A civilian setting is a study that describes civilian patients treated by non-military personnel, such as EMS personnel, doctors, laymen, fire constables, and police enforcement in a civilian environment. A military setting is a study that describes patients (military personnel and civilians) treated by military personnel (medics, soldiers, and military hospitals) in a warfare environment. A tourniquet is a constricting or compression device used to control arterial and venous blood flow to a portion of an extremity for a period of time. An improvised tourniquet is a tourniquet made from materials originally not intended to be used as a tourniquet, such as belts and clothes. A commercial tourniquet is a commercially available product made solely to be used as a tourniquet.

### Patient and Public Involvement

No patients were involved in the design of the study. The study was not commissioned, and there was no public involvement.

## Results

### Included Studies

**Figure 1.**
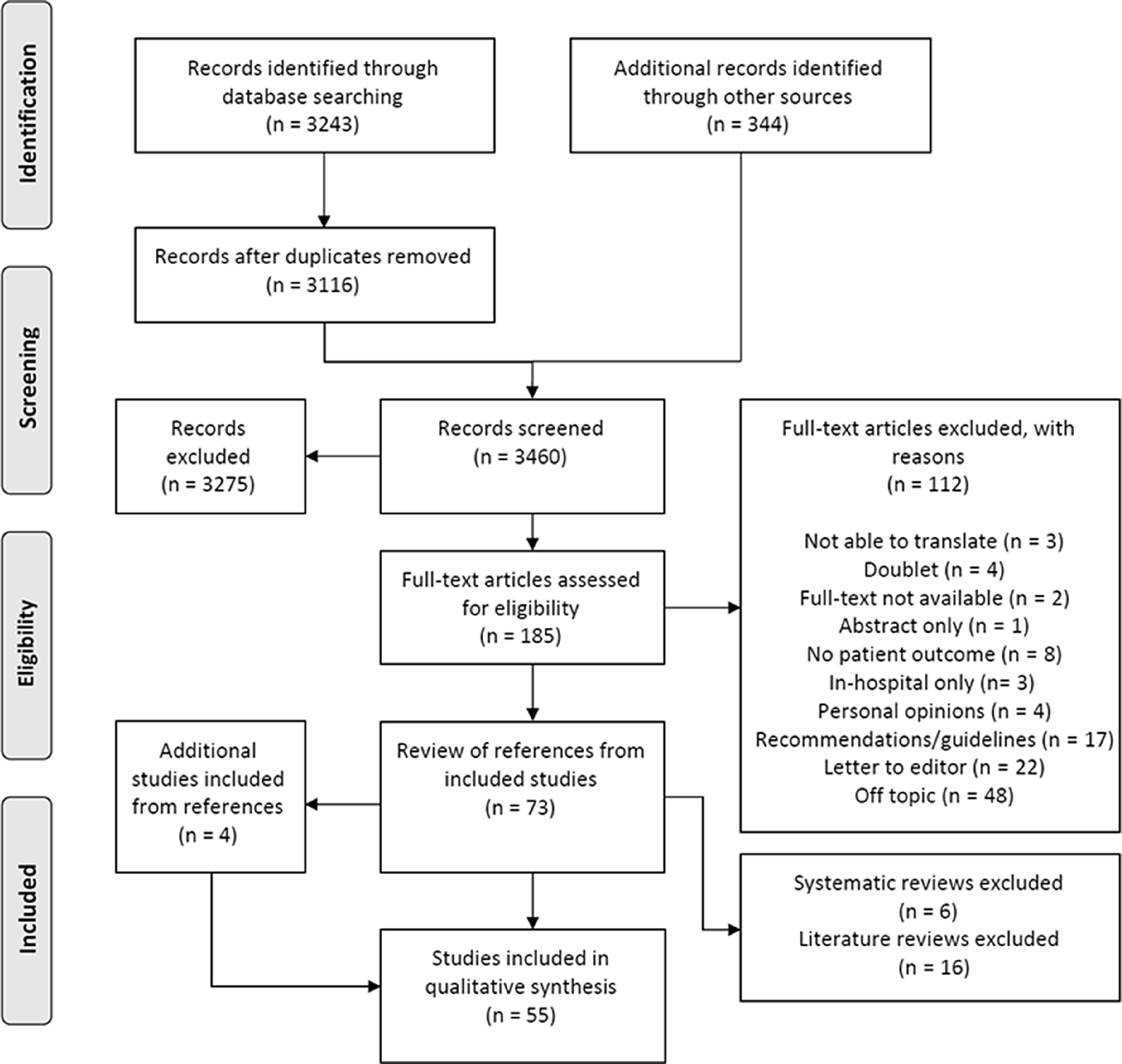
PRISMA Flow Diagram of the Selection of Included Studies.

After duplicate removal, 3,116 studies were identified. Title and abstracts were screened for eligibility, and the remaining 185 studies were screened for eligibility through full-text review. Finally, 112 studies were excluded. A total of 344 on-going studies were identified at clinicaltrials.gov, WHO ICTRP, and PROSPERO. This resulted in 73 eligible studies. Another four studies were included after reviewing the reference lists of the systematic reviews and literature reviews. Two of the studies were not found in the original search, one because it was published before 2000^11^ and the other because tourniquet was not mentioned in the title or abstract.^12^ Two more studies were originally excluded from the title and abstract review, but were included after reading the full-text from references: one case report^13^ and one literature review.^14^ After excluding all non-primary literature such as literature reviews^14–29^ and systematic reviews,^30–35^ a total of 55 studies were included in this systematic review. The process from search result to inclusion is illustrated in Figure 1.

### Quality of Evidence

All included studies were published from January 1, 2000 through January 10, 2019 and consisted of 15 civilian retrospective observational studies,^36–50^ two civilian case series,^51,52^ 12 civilian case reports,^13,53–63^ six prospective observational military studies,^64–69^ 16 military retrospective observational studies,^11,12,70–83^ two military case series,^84,85^ one military case study,^86^ and one military case report.^87^ The included studies are summarized in Table 1. None of the studies reported on all of the criteria, with 36%-87% of the criteria being met. No published high-quality studies were found, all published studies had very low evidence according to GRADE, mostly due to their observational character and small sample size.^10^ A detailed quality appraisal is available as Appendix III (available online only).

**Table 1.** An Overview and Summary of the Included Studies, Excluding Case Reports

Civilian Studies				
Author Country Year Ref. No.	Study Type	Study Size Patients with TQ	TQ Time	Outcomes
Kalish USA 2008^37^	OR	11	75 min (avg.)	Survival rate: 90.9% Fasciotomy: 18.2%
Rtshiladze Australia 2011^51^	CS	2		Survival rate: 100%
Passos Canada 2014^50^	OR	8		Survival rate: 100% Transfusions: 4 units (avg.) Compartment syndrome: 0%
Callaway USA 2015^52^	CS	4	70.5 min (avg.)	Survival rate: 100% Transfusions: 4.67 units (avg.) Amputation: 0% Lasting nerve palsy: 0% Fasciotomy: 75%
Inaba USA 2015^38^	OR	87	103.2 min (avg.)	Survival rate: 100% Transfusions: 4.1 units (avg.) Amputation: 1.1% Compartment syndrome: 1.1% Infection: 2.3
King USA 2015^49^	OR	27		Survival rate: 100%
Kue USA 2015^36^	OR	98	15 min (avg.)	Survival rate: 89.8%
Leonard USA 2015^42^	OR	61	21 min (median)	Survival rate: 90.2% Transfusions: 7.4 units (avg.) Lasting nerve palsy: 0% Compartment syndrome: 0% Fasciotomy: 6.6% Infection: 6.6%
Ode USA 2015^39^	OR	24	72 min (avg.)	Survival rate: 87.5%
Schroll USA 2015^40^	OR	197		Survival rate: 97% Transfusions: 3.7 units (avg.) Compartment syndrome: 8.6% Infection: 8.6%
Zietlow USA 2015^41^	OR	73		Survival rate: 98.6%
Scerbo USA 2016^43^	OR	105		Survival rate: 93.3% Transfusions: 2 units (avg.) Amputation: 0% Lasting nerve palsy: 0% Compartment syndrome: 1.9% Fasciotomy: 26.7%
Ballas France 2017^44^	OR	4		Survival rate: 100%
Scerbo USA 2017^45^	OR	306		Survival rate: 93.1% Transfusions: 3 units (avg.) Compartment syndrome: 1.6%
Duignan USA 2018^46^	OR	5		Survival rate: 80%
Teixeira USA 2018^47^	OR	181	77.3 min (avg.)	Survival rate: 96.1% Transfusions: 5 units (avg.) Infection: 13.8%
Smith USA 2019^48^	OR	238	34.9 min (avg.)	Survival rate: 91.2% Transfusions: 2.2 units (avg.) Compartment syndrome: 5.9% Fasciotomy: 11.3% Infection: 8%
**Military Studies**				
Lakstein Israel 2003^72^	OR	91	83 min (avg.)	
Pilgram - Larsen Norway 2004^11^	OR	18		Survival rate: 83%
Mucciarone USA 2006^86^	CSt	2		Survival rate: 100% Amputation: 0%
Brodie UK 2007^73^	OR	70		Survival rate: 87% Compartment syndrome: 2.9%
Holcomb USA 2007^71^	OR	1		Survival rate: 0%
Beekley USA 2008^74^	OR	67	70 min (avg.)	Survival rate: 96% Transfusions: 8.8 units (avg.) Lasting nerve palsy: 0% Amputation: 0%
Dayan Israel 2008^84^	CS	5	795 min (avg.)	Survival rate: 100% Lasting nerve palsy: 0% Amputation: 20% Compartment syndrome: 20% Fasciotomy: 20%
Kragh USA 2008^64^	OP	232	78 min (avg.) 60 min (median)	Survival rate: 86.6% Lasting nerve palsy: 0.4% Amputation: 0.4% Compartment syndrome: 0% Fasciotomy: 41.4%
Nelson USA 2008^85^	CS	3		Survival rate: 0% Transfusions: 10.5 units (avg.)
Tien USA 2008^65^	OP	6		Survival rate: 100%
Clasper UK 2009^75^	OR	-	60 min (median)	Infection: 18 patients
Kragh USA 2009^66^	OP	232		Survival rate: 86.6% Lasting nerve palsy: 0.4% Amputation: 0%
Brown UK 2010^76^	OR	23		Infection: 52.2%
Gerhardt USA 2011^77^	OR	8		Survival rate: 75%
Kotwal USA 2011^12^	OR	66		Survival rate: 94%
Kragh USA 2011^67^	OP	499		Survival rate: 86.6%
Kragh USA 2011^68^	OP	499		Lasting nerve palsy: 0.2% Fasciotomy: 29.7%
Cheng China 2012^78^	OR	7		Survival rate: 29%
Kragh USA 2013^69^	OP	727		Survival rate: 88%
Kragh USA 2015^79^	OR	1272		Survival rate: 92%
Kragh USA 2015^80^	OR	720		Survival rate: 88% Transfusions: 12 units (avg.)
Dunn USA 2016^81^	OR	24		Survival rate: 96%
Dunn USA 2016^82^	OR	6		Survival rate: 83%
Shlaifer Israe 2017^83^	OR	90		Survival rate: 88% Lasting nerve palsy: 3.3% Fasciotomy: 8.9%
Staudt USA^70^	OR	1105		

Abbreviations: CS, case series; CSt, case study; OP, observational prospective; OR, observational retrospective; TQ, tourniquet.

Some of the studies included tourniquets applied in the emergency department alongside tourniquets applied in the prehospital setting. When the two groups were clearly differentiated, only the report on the prehospital tourniquet application group was reported.

### Mortality

**Figure 2.**
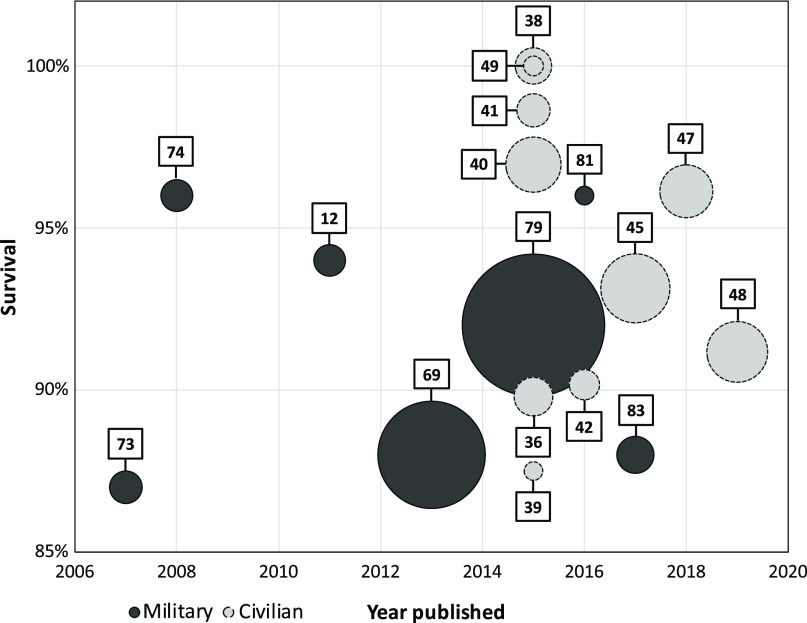
Studies Reporting on Mortality, Excluding Case Reports and Studies with Less Than 20 Participants. Note: Circle size indicates the number of patients treated with tourniquet. The center of the circle indicates the survival rate. Each study is represented by its reference number.

Fifty studies reported mortality in patients treated with tourniquet. Twenty-seven of these were either case reports or had less than 20 participants and were not included in assessing the primary outcome.^11,13,37,44,46,50–63,65,71,77,78,84–87^ Twenty-three studies were included to assess mortality.^12,36,38–43,45,47–49,64,66,67,69,73,74,79–83^ Some of the studies reported data from the same population/database. Figure 2 shows data extracted from the latest published study to report mortality rate, consisting of ten civilian and seven military studies.^12,36,38–42,45,47–49,69,73,74,79,81,83^ The figure shows the reported survival, publication year, and sample size in each of these included studies. The studies reported a survival rate between 87%-100%. None of the studies were randomized controlled studies, and studies with comparison groups were hampered by unclear indications for tourniquet placement and bias due to a risk of more severe injuries in the tourniquet group.

Overall, the military studies reported similar mortality rates in patients treated with tourniquets and patients who were not, despite the patients in the tourniquet group being more severely injured. Early application before the onset of shock was strongly associated with increased survival. One study^67^ found that 96% of patients survived when the tourniquet was placed before the onset of shock compared to four percent of patients who received a tourniquet later. Patients treated with prehospital tourniquet (89%) also had higher survival rates than those who received a tourniquet upon arrival at the hospital (76%-78%).^66,67^

Overall, studies indicated a survival benefit for patients treated with tourniquets in a civilian setting. Patients treated with prehospital tourniquet had a lower incidence of shock when arriving at the hospital compared to those who were not treated with tourniquet.^48^ One study reported that death from hemorrhagic shock was more frequent in patients who received a tourniquet in-hospital (14%) compared to those who received a prehospital tourniquet (three percent).^45^ Most studies reported an average tourniquet application time less than two hours.

### Blood Transfusions

Seventeen studies reported on blood transfusions in patients treated with tourniquets: nine civilian observational studies,^38,40,42,43,45,47,48,50,52^ three military observational studies,^74,80,85^ and five civilian case reports.^53,54,59,62,63^ In the majority of the civilian studies with a control group, patients with a prehospital tourniquet received fewer blood products than patients not treated with tourniquets or if the tourniquets were placed in-hospital. In the military studies, the tourniquet patients received more blood products than their respective control groups. In one study,^80^ the difference between the study groups was significant, but most of the patients in the tourniquet group also had non-extremity injuries, which may have affected the data.

### Other Complications

Thirty-five of the studies reported on complications associated with the use of tourniquets, 21 in the civilian setting and 14 in the military setting; 10 of these were case reports. Fifteen studies reported on nerve palsy associated with the use of tourniquet.^36,40,42,43,48,52,64,66–68,72–74,83,84^ Six civilian studies reported on nerve palsies attributed to the use of tourniquet in 18 of 465 patients. One study found that tourniquets were not associated with nerve palsies when comparing two groups of patients (127 treated with tourniquet versus 77 patients not treated with tourniquet).^48^ Neurological complications seemed to be few and most were transient.

Nine studies reported on amputations as a complication of the use of a tourniquet, one of which was a case report. Four of the studies were civilian^38,43,52,62^ and five were military.^64,66,74,84,86^ To summarize, very few amputations were solely due to the use of tourniquets, but the few casuistic amputations related to tourniquet use per se were mainly related to improvised tourniquets and prolonged tourniquet application time.

A need for fasciotomy and/or compartment syndrome in patients treated with tourniquet was reported in 17 studies, three of which were case reports.^37,38,40,42,43,45,48,50,52,55,58,64,68,73,83,84,87^ Fasciotomy seemed to be a frequent treatment modality after the use of tourniquets, and compartment syndrome was not infrequent if fasciotomy was not performed. Tourniquet application time greater than two hours seemed to increase fasciotomy rates.^68^

Seven studies reported on infections associated with tourniquet use: five civilian^38,40,42,47,48^ and two military studies.^75,76^ The civilian studies had a total of 764 patients with tourniquets, 67 of which were reported to have a wound infection (nine percent). Tourniquets were not associated with a difference in secondary infection rates compared to the non-tourniquet groups.

## Discussion

Currently available knowledge did not allow for a systematic review with meta-analyses. Most studies were cohorts and case reports, and no randomized controlled studies were found. Thus, a descriptive synthesis was performed. Most studies indicated that early application of a tourniquet before the onset of shock increased survival in patients bleeding from an extremity. The difference in survival between patients treated with tourniquets and those without was low, possibly because patients treated with tourniquets were more severely injured. This suggests that tourniquets may play a central role in saving the lives of patients with non-controllable extremity bleeding. The studies also showed that patients with isolated extremity bleeding required fewer blood transfusions when treated with tourniquets, and that the adverse effects of tourniquet use were few and predictable.

The studies were dominated by military studies before 2012 and civilian studies after 2015. This may be due to the civilian focus on implementing military guidelines in mass-casualty events. When comparing the military studies with the civilian studies, differences in the mechanism of injury were found. However, the indication for the use of tourniquets was similar: uncontrollable hemorrhage from the extremities. The tourniquet application time was considered to be an important factor. The application time was similar between the military and civilian studies, but considerably shorter in civilian urban areas.

Numerous complications from the use of tourniquets have been described. They seemed to be infrequent and many resolved. However, in a life-over-limb situation, the risk seemed negligible. When the tourniquets were used correctly, the reports indicated hardly any risk of amputation due to the tourniquet. Improvised tourniquets seemed to be less effective than commercial tourniquets and may increase the risk of venous stasis and paradoxical bleeding.

The findings in this study correspond to those in the systematic reviews identified in the present search.

## Limitations

All of the findings in this review have low to very low strength of evidence due to the observational character of the included studies. Most studies are biased, as patients who died before arriving at the hospital are not included in the analyzed databases. Despite the extensive search, there is a risk that relevant studies may have been missed. Three (Chinese and Cyrillic) studies were excluded due to language.

## Conclusion

Despite low level of evidence in the studies identified, the studies consistently indicated that the use of a tourniquet was associated with increased survival in uncontrollable extremity bleeding in a civilian setting. The civilian and military studies reported similar findings and treatment efficiency, though military studies tended to have longer application times. Complications to tourniquet use seemed to be tightly related to application time, and application times less than two hours seemed to be reasonably safe in previously healthy patients. Application times in urban civilian settings were short.

## Supplementary material

For supplementary material accompanying this paper visit https://doi.org/10.1017/S1049023X20001284v. click here to view supplementary material
