# Supplementary material for: Prehospital Tourniquets in Civilians: A Systematic Review
Source: Prehosp Disaster Med. 2020 Nov 3;36(1):86–94. doi: 10.1017/S1049023X20001284 (PMC7844612; doi:10.1017/S1049023X20001284)
Supplement: Supplementary file 1 [file S1049023X20001284sup.zip › S1049023X20001284sup002.pdf]

Database: Ovid MEDLINE(R) and Epub Ahead of Print, In-Process & Other Non-Indexed Citations and Daily <1946 to January 08, 2019>
Search date: 2019-01-10

1 Tourniquets/ (3617)
2 tourniquet*.ti,ab. (5828)
3 1 or 2 (6874)
4 exp "Wounds and Injuries"/ (853172)
5 (wound* or trauma* or injur*).ti,ab. (1067419)
6 4 or 5 (1550568)
7 exp Extremities/ or (extremit* or limb or limbs or groin or leg or legs or thigh or thighs or axilla or armpit or arm or arms or forearm or forearms).ti,ab. (746162)
8 3 and 6 and 7 (1167)
9 "out of hospital".ti,ab. (8980)
10 outside-hospital*.ti,ab. (1380)
11 (prehospital or pre-hospital or on-site or accident*).ti,ab. (132514)
12 civilian*.ti,ab. (10876)
13 Military Medicine/ (28885)
14 Military Personnel/ (37356)
15 (armed forces or army).ti,ab. (18232)
16 (military or soldier*).ti,ab. (48510)
17 exp Ambulances/ (8150)
18 ambulance*.ti,ab. (9451)
19 exp Emergency Treatment/ (112432)
20 emergenc*.ti,ab. (328500)
21 Emergency Medicine/ or Emergency Medical Services/ (51074)
22 Allied Health Personnel/ (11259)
23 paramedic*.ti,ab. (7227)
24 (combat or battle* or collision* or catastrophe* or disaster* or war).ti,ab. (130628)
25 Terrorism/ (4963)
26 terror*.ti,ab. (7702)
27 or/9-26 (766086)
28 3 and 27 (758)
29 8 or 28 (1618)
30 Animals/ (6328115)
31 Humans/ (17481585)
32 30 not (30 and 31) (4501407)
33 29 not 32 (1347)

Database: Embase 1974 to 2019 January 09
Search date: 2019-01-10

1 tourniquet/ (5499)
2 tourniquet*.ti,ab. (6800)
3 1 or 2 (8241)
4 exp injury/ (1925988)
5 exp wound/ (233603)
6 (wound* or trauma* or injur*).ti,ab. (1344024)
7 4 or 5 or 6 (2414066)
8 exp limb/ (596194)
9 (extremit* or limb or limbs or groin or leg or legs or thigh or thighs or axilla or armpit or arm or arms or forearm or forearms).ti,ab. (752038)
10 8 or 9 (1084799)
11 3 and 7 and 10 (1727)

12 "out of hospital".ti,ab. (14835)
13 outside-hospital*.ti,ab. (3305)
14 (prehospital or pre-hospital or on-site or accident*).ti,ab. (170246)
15 civilian*.ti,ab. (12033)
16 military medicine/ (18763)
17 soldier/ (27428)
18 army/ (14721)
19 (armed forces or army).ti,ab. (17333)
20 (military or soldier*).ti,ab. (53830)
21 exp ambulance/ (12647)
22 ambulance*.ti,ab. (13729)
23 emergency medicine/ (37924)
24 exp emergency treatment/ (236999)
25 emergenc*.ti,ab. (446742)
26 exp paramedical personnel/ (443669)
27 paramedic*.ti,ab. (10265)
28 battle injury/ (4100)
29 war/ (28603)
30 terrorism/ (8508)
31 terror*.ti,ab. (9144)
32 (combat or battle* or collision* or catastrophe* or disaster* or war).ti,ab. (131035)
33 or/12-32 (1406935)
34 3 and 33 (1226)
35 11 or 34 (2522)

Database: Cochrane Library
Search date: 2019-01-10

#1 MeSH descriptor: [Tourniquets] explode all trees 464
#2 tourniquet*:ti,ab,kw in Cochrane Reviews 5
#3 tourniquet* in Cochrane Protocols, Trials 1510
#4 #1 or #2 or #3 1515

Database: Epistemonikos
Search date: 2019-01-10

Tourniquet 155

Database: PROSPERO
Search date: 2019-01-10

Tourniquet 32

Database: clinicaltrials.gov
Search date: 2019-01-10

Tourniquet 297

Database: WHO ICTRP
Search date: 2019-01-10

Tourniquet 254
