# Supplementary material for: Prehospital Tourniquets in Civilians: A Systematic Review
Source: Prehosp Disaster Med. 2020 Nov 3;36(1):86–94. doi: 10.1017/S1049023X20001284 (PMC7844612; doi:10.1017/S1049023X20001284)
Supplement: Supplementary file 1 [file S1049023X20001284sup.zip › S1049023X20001284sup003.pdf]

Ref. Number	Author	Year published	Patients with TQ	TQ applied	1. Did the study address a clearly focused issue?	2. Was the cohort recruited in an acceptable way?	3. Was the exposure accurately measured t o minimise bias?	4. Was the outcome accurately measured to minimise bias?	5a. Have the authors identified all important confounding factors?	5b. Have they taken account of the confounding factors in the design and/or analysis?	6a. Was the follow up of subjects complete enough?	6b. Was the follow up of subjects long enough?	7. What are the results of this study?	8. How precise are the results?	9. Do you believe the results?	10. Can the results be applied to the local population?	11. Do the results of this study fit with other available evidence?	12. What are the implications of this study for practice?
**Civilian**																			
37	Kalish	2008	11	11	y	y	y	y	n	n	y	NR	See manuscript	See manuscript	y	y	y	n
51	Rtshiladze	2011	2	3	y	y	n	u	n	n	y	NR	See manuscript	See manuscript	y	n	y	n
50	Passos	2014	8	8	y	y	y	y	y	y	y	NR	See manuscript	See manuscript	y	y	y	n
52	Callaway	2015	4	4	y	y	u	u	n	n	y	NR	See manuscript	See manuscript	y	y	y	n
38	Inaba	2015	87	87	y	y	y	y	n	y	y	NR	See manuscript	See manuscript	y	y	y	n
49	King	2015	27	27	y	y	y	y	n	y	y	NR	See manuscript	See manuscript	y	y	y	n
36	Kue	2015	98	98	y	y	y	y	n	y	y	NR	See manuscript	See manuscript	y	y	y	n
42	Leonard	2015	61	62	y	y	y	y	n	n	y	NR	See manuscript	See manuscript	y	y	y	n
39	Ode	2015	24	25	y	y	y	y	n	y	y	NR	See manuscript	See manuscript	y	y	y	n
40	Schroll	2015	197	-	y	y	y	y	n	y	y	NR	See manuscript	See manuscript	y	y	y	u
41	Zietlow	2015	73	77	y	y	y	y	n	n	y	NR	See manuscript	See manuscript	y	y	y	n
43	Scerbo	2016	105	115	y	y	y	y	n	y	y	NR	See manuscript	See manuscript	y	y	y	u
44	Ballas	2017	4	-	y	y	y	y	n	n	y	NR	See manuscript	See manuscript	y	y	y	n
45	Scerbo	2017	306	326	y	y	y	y	n	y	y	NR	See manuscript	See manuscript	y	y	y	u
46	Duignan	2018	5	5	y	y	y	y	n	y	y	NR	See manuscript	See manuscript	y	y	y	n
47	Teixeira	2018	181	-	y	y	y	y	n	y	y	NR	See manuscript	See manuscript	y	y	y	u
48	Smith	2019	238	-	y	y	y	y	n	y	y	NR	See manuscript	See manuscript	y	y	y	u
**Military**																			
72	Lakstein	2003	91	110	y	y	y	y	n	y	y	NR	See manuscript	See manuscript	y	y	y	n
11	Pilgram - Larsen	2004	18	-	y	y	y	y	n	y	y	NR	See manuscript	See manuscript	y	n	u	n
86	Mucciarone	2006	2	3	y	y	u	u	n	n	y	NR	See manuscript	See manuscript	y	n	y	n
73	Brodie	2007	70	107	y	y	y	y	n	n	y	NR	See manuscript	See manuscript	y	y	y	n
71	Holcomb	2007	1	-	y	y	y	y	n	n	y	NR	See manuscript	See manuscript	y	y	y	n
74	Beekley	2008	67	80	y	y	y	y	n	y	y	NR	See manuscript	See manuscript	y	y	u	n
84	Dayan	2008	5	5	y	y	u	u	n	n	y	NR	See manuscript	See manuscript	y	n	y	n
64	Kragh	2008	232	428	y	y	y	y	n	y	y	NR	See manuscript	See manuscript	y	y	y	u
85	Nelson	2008	3	5	y	y	y	u	n	n	y	NR	See manuscript	See manuscript	y	n	y	n
65	Tien	2008	6	8	y	y	y	y	n	n	y	NR	See manuscript	See manuscript	y	y	y	n
75	Clasper	2009	-	-	y	y	y	y	n	y	y	NR	See manuscript	See manuscript	y	y	y	n
66	Kragh	2009	232	428	y	y	y	y	n	y	y	NR	See manuscript	See manuscript	y	y	y	u
76	Brown	2010	23	-	y	y	y	y	n	y	y	NR	See manuscript	See manuscript	y	y	y	n
77	Gerhardt	2011	8	-	y	y	y	y	n	y	y	NR	See manuscript	See manuscript	y	y	y	n
12	Kotwal	2011	66	89	y	y	y	y	n	y	y	NR	See manuscript	See manuscript	y	y	y	n
67	Kragh	2011	499	862	y	y	y	y	n	y	y	NR	See manuscript	See manuscript	y	y	y	y
68	Kragh	2011	499	862	y	y	y	y	n	n	y	NR	See manuscript	See manuscript	y	y	y	y
78	Cheng	2012	7	-	y	y	y	y	n	y	y	NR	See manuscript	See manuscript	y	y	y	n
69	Kragh	2013	727	1212	y	y	y	y	n	n	y	NR	See manuscript	See manuscript	y	y	y	y
79	Kragh	2015	1272	-	y	y	y	y	n	y	y	NR	See manuscript	See manuscript	y	y	y	y
80	Kragh	2015	720	-	y	y	y	y	n	y	y	NR	See manuscript	See manuscript	y	y	y	y
81	Dunn	2016	24	-	y	y	y	y	n	y	y	NR	See manuscript	See manuscript	y	y	y	n
82	Dunn	2016	6	-	y	y	y	y	n	y	y	NR	See manuscript	See manuscript	y	y	y	n
83	Shlaifer	2017	90	119	y	y	y	y	n	y	y	NR	See manuscript	See manuscript	y	y	y	n
70	Staudt	2018	1105	-	y	y	y	y	n	y	y	NR	See manuscript	See manuscript	y	y	y	n
